# The effect of food composition on serum testosterone levels after oral administration of Andriol® Testocaps®

**DOI:** 10.1111/j.1365-2265.2007.02781.x

**Published:** 2007-04-01

**Authors:** Peter G Schnabel, Wilma Bagchus, Holger Lass, Torben Thomsen, T B Paul Geurts

**Affiliations:** * Department of Clinical Pharmacology and Kinetics NV Organon, Oss, the Netherlands; † Department of Bioanalytics, Organon Development GmbH Waltrop, Germany; ‡ Pharm PlanNet Contract Research GmbH Mönchengladbach, Germany; § International Medical Services NV Organon, Oss, the Netherlands

## Abstract

**Objective:**

Andriol® Testocaps® is a new oral formulation of testosterone undecanoate (TU) for treatment of hypogonadism. As TU is taken up by the intestinal lymphatic system, both the presence and the composition of food influence the absorption. The aim of this study was to investigate the effect of food composition on the pharmacokinetics of oral TU.

**Design:**

An open-label, single-centre, four-way crossover study. With a washout period of 6–7 days, 80 mg TU was administered in the morning 5 min after consuming each of four different meals in a randomized order (A: 230 kcal, 0·6 g lipid; B: 220 kcal, 5 g lipid; C: 474 kcal, 19 g lipid; D: 837 kcal, 44 g lipid).

**Patients:**

Twenty-four postmenopausal volunteers.

**Measurements:**

Serial blood samples were collected until 24 h after dosing to determine testosterone and dihydrotestosterone (DHT) by gas chromatography-mass spectroscopy (GC-MS).

**Results:**

The bioavailability of testosterone after a low-calorie meal containing 0·6 g lipid or 5 g lipid was relatively low, the area under the concentration–time curve (AUC_0–tlast_) for testosterone being 30·7 and 43·5 nmol h/l, respectively. The bioavailability of testosterone after a meal containing 19 g lipid was considerably higher (AUC_0–tlast_ = 146 nmol h/l), whereas increasing the lipid content to 44 g lipid did not further increase the bioavailability of testosterone (AUC_0–tlast_ = 154 nmol h/l).

**Conclusion:**

Approximately 19 g of lipid per meal efficiently increases absorption of testosterone from oral TU. Therefore, coadministration with a normal rather than a fatty meal is sufficient to increase serum testosterone levels when using oral TU.

## Introduction

Testosterone replacement therapy is intended to restore normal serum testosterone levels in patients with hypogonadal disorders. Although testosterone itself is absorbed well after oral administration, it is rapidly metabolized in the intestinal wall and during its first pass through the liver, thereby inactivating approximately 98% of the amount absorbed. As a consequence, oral administration of pure, crystalline testosterone does not increase serum testosterone levels sufficiently. Such preparations are not therefore suitable for oral administration in hypogonadal disorders, as they do not result in sustained physiological serum testosterone levels.^1^,^2^ Various solutions to this problem have been developed, including injectable and transdermal routes of administration as well as sophisticated systems for oral administration that circumvent hepatic first-pass metabolism.^3^ Oral testosterone undecanoate (Andriol® Testocaps®) is the only oral form of testosterone replacement therapy that restores testosterone levels within the normal range and is available in more than 80 countries. It consists of a solution of testosterone undecanoate (TU) in an oily vehicle, contained in a soft gelatin capsule.^4^ In contrast to crystalline testosterone, TU dissolved in a lipophilic solvent significantly enhances absorption.^1^ De-esterification of TU to produce testosterone and 5α-reduction to produce dihydro-TU (DHTU) take place rapidly in the intestinal wall as well as in the peripheral circulation.^5^–^7^

Many studies have shown that food can have a marked effect on drug pharmacokinetics by increasing, decreasing and/or delaying drug absorption.^8^,^9^ A study with oral TU in men has suggested that, if taken with a meal, the TU molecules are included in chylomicrons. As a result, a significant part of the administered TU bypasses the liver and gains access to the peripheral circulation through the intestinal lymphatic system, thereby further increasing serum testosterone levels.^10^ Recently, the effect of food on the bioavailability of oral TU was investigated in more detail. In a single-dose, randomized crossover study, the effect of a standardized meal on the pharmacokinetics of oral TU was compared with administration in a fasting state. It was found that in the fasting state hardly any TU was absorbed and oral administration of TU with food dramatically enhanced the bioavailability of TU. It was concluded that for optimal absorption, oral TU capsules must be taken with food.^11^ However, as this study contained only one standardized type of meal, the optimal amount of food and which meal composition would be optimal for adequate absorption of TU after oral administration could not be determined. In the absence of clear guidelines, the advice has often been to take oral TU capsules with a ‘fatty meal’ to ensure optimal absorption of the active ingredient.^12^ From the patient's perspective, this issue is relevant because twice daily administration of the capsules with a ‘fatty meal’ is undesirable.

The aim of the current study was to investigate the effect of food composition on the bioavailability of oral TU.

## Methods

### Pharmaceutical formulation

Andriol® Testocaps® are oval, orange, soft gelatin capsules containing 40 mg TU in castor oil with propylene glycol laurate [60 : 40 (w/w)], glycerin and sunset yellow (E110). Within 5 min of each test meal, the subjects received two capsules (total 80 mg) of oral TU.

### Study centre

The clinical part of the study was performed at Pharm PlanNet Contract Research GmbH, D-41061 Mönchengladbach, Germany. The study protocol was approved by the Independent Ethics Committee of the Physicians Chamber North-Rhine (Ärztekammer Nordrhein), Düsseldorf, Germany. The trial was conducted in compliance with the currently accepted revision of the Declaration of Helsinki, the guidelines for Good Clinical Practice and Principles of Good Laboratory Practice (GLP).

### Subjects

A total of 24 healthy, postmenopausal women, aged 45–65 years and with a body mass index (BMI) of 18–30 kg/m^2^, participated in this study. To be considered for inclusion subjects had to provide written informed consent before screening evaluations, and have a pretrial screening total testosterone level < 2·5 nmol/l, smoking < 10 cigarettes/day, be in good age-appropriate physical and mental health, have a normal cervical smear performed within the past 12 months and a normal mammogram performed within the past 24 months. Subjects had to refrain from all use of grapefruit juice and caffeine and other methylxanthines (e.g. coffee, tea, cola or chocolate) from 48 h prior to each dosing until the last pharmacokinetic blood sampling, 24 h after each dosing. Subjects were excluded from the study if they had a history of sensitivity to TU or chemically related substances or used drugs known to alter oestrogen metabolism or affect cytochrome P450 enzymes, except after an appropriate washout period. Subjects were excluded if they had signs of severe acne or hirsutism, or abnormal blood pressure or heart rates, were HIV or hepatitis B/C positive. Subjects were also excluded if they had a history of significant allergic or other diseases, including malignancies, drug, alcohol or solvent abuse, had donated blood or participated in an investigative drug trial within 90 days before the start of this study or during the study. Subjects were not allowed to use any prescription or over-the-counter medication for 7 days before the first dose until the last pharmacokinetic sampling except for sporadic use of paracetamol.

### Clinical study design and blood sampling

The study was an open-label, single-centre, four-period, crossover design with a washout of 6–7 days between each treatment and the follow-up. For logistic reasons the trial was performed in two cohorts of 12 subjects. Volunteers were randomized to receive one of four sequences of therapy. The sequences were characterized by the Latin square sequence of the meals, which were either ABCD, BDAC, CADB or DCBA. Volunteers fasted overnight (for at least 10 h) and in the morning they received a single oral dose of 80 mg TU (two capsules) with 150 ml water, exactly 5 min after the end of the A, B, C or D meal, which they had consumed over a period of 10 min (A, B), 15 min (C) or 20 min (D). Thereafter, subjects fasted for another 4 h (but after 1 h they were allowed to drink water *ad libitum*), after which a lunch was served; 10 h after breakfast, dinner was served and in the evening a snack was served. Serial blood samples were taken before dosing and at 1, 2, 3, 4, 5, 6, 7, 8, 9, 10, 11, 12, 14, 16, 20 and 24 h after dosing with oral TU for determination of serum TU, testosterone, DHTU and dihydrotestosterone (DHT). Immediately after collection, blood was processed to serum, divided over two tubes and stored in a freezer.

In this study the effects of four different meal compositions on the pharmacokinetics of oral TU were investigated:

Meal A (‘fat-free’ meal) consisted of yoghurt (175 ml, 0·16% lipids), 250 ml milk (0·16% lipids) and 20 g sugar. The calculated total amount of lipids was 0·7 g and the calculated caloric value was 215 kcal.
Meal B (‘low-fat’ meal) consisted of yoghurt (175 ml, 1·5% lipids), 250 ml milk (1·02% lipids) and 5 g sugar. The calculated total amount of lipids was 5 g and the calculated caloric value was 215 kcal.
Meal C (‘normal’ meal) contained two bread rolls, one slice of cheese (20 g, 40% lipids), one slice of ham (25 g), 20 g jam, 10 g margarine and two cups caffeine-free coffee (300 ml). The calculated total amount of lipids was 20 g and the calculated caloric value was 430 kcal.
Meal D (‘fatty’ meal) consisted of two eggs fried with 5 g butter, two strips of bacon, two slices of toast with 7·5 g butter, 4 oz (113 g) hash brown potatoes and 8 oz (226 ml) of whole milk. The calculated total amount of lipids was 50 g and the calculated caloric value was 850 kcal.

A spare meal of types A, B, C and D was collected, frozen and send to Analytico, Heerenveen, the Netherlands, for chemical analysis of the contents of the meal regarding lipid content, carbohydrates, proteins and caloric value.

The lunch consisted of cooked slices of turkey with a thick sauce of mushrooms and green peppers, rice, one medium-sized apple, lettuce and tomato with yoghurt dressing, yoghurt with fruit, totalling 900 kcal, 28 g lipids. The evening dinner consisted of two slices of dark bread, 20 g margarine, one slice of cheese, one slice of cold meat, rice salad with tomato and onions, totalling 746 kcal, 29 g lipids. In the evening subjects were allowed an evening snack consisting of one apple (81 kcal, 0·9 g lipids). Subjects were required to refrain from consuming grapefruit juice, caffeine and other methylxanthines (e.g. coffee, tea, cola or chocolate) from 48 h before dosing until the last pharmacokinetic sampling of each dose.

### Analytical design

TU and DHTU concentrations were determined using a validated liquid chromatographic (LC) assay with mass spectrometry (MS) detection after solid-phase extraction. The extracts were quantified by LC-MS using electrospray ionization in multireaction monitoring (MRM) mode. The analysis of testosterone and DHT concentrations in serum were assayed using a validated GC assay with MS detection after solid-phase extraction, derivatization and liquid–liquid extraction with *n*-hexane.

Each analytical series of TU, DHTU, testosterone and DHT consisted of patient samples, eight calibration samples (in duplicate), three or four quality control (QC) samples (in triplicate), two blank internal standard samples (in duplicate) and at least one blank serum (TU and DHTU only) and one blank water sample. The lower limit of quantification was 0·438 nmol/l for TU, 0·436 nmol/l for DHTU, 0·347 nmol/l for testosterone and 0·344 nmol/l for DHT. The interassay coefficient of variation for the QC samples was in the range 4·8–11·0% for TU, 4·0–30·1% for DHTU, 2·3–5·8% for testosterone and 4·5–11·9% for DHT. The accuracy of the QC samples was in the range 93·9–100·3% for TU, 103·0–114·2% for DHTU, 99·2–102·9% for testosterone and 91·7–102·2% for DHT. Bioanalysis was performed at the Bioanalytics Section of the Department of Metabolism and Kinetics, Organon Development GmbH, Waltrop, Germany (now Department of Bioanalytics, Organon Development GmbH, Waltrop) with validated methods, and in compliance with GLP principles of the OECD.

### Pharmacokinetic parameters

Pharmacokinetic parameters were calculated by subject and by treatment from serum concentrations of TU, DHTU, testosterone and DHT. Maximum serum concentrations (*C*_max_) and time to *C*_max_ (*t*_max_) were taken from the measured serum concentration data. The area under the concentration–time curve from zero to *t*_last_ (AUC_0–tlast_) was calculated by means of the linear trapezoidal rule, where *t*_last_ represented the last time point with a measurable concentration. Descriptive statistics for the concentrations were only calculated if at least two-thirds of the concentrations by time point were greater than or equal to the lower limit of quantification (LLOQ). If that was the case, then the concentrations indicated as < LLOQ were replaced by 0·5 × LLOQ for the calculation of the descriptive statistics.

### Statistical analysis

Bioequivalence testing was performed to compare the pharmacokinetics of oral TU under different food conditions, where meal C treatment was taken as reference treatment and meals A, B and D as test treatments. Meal B treatment was also compared to meal A treatment.

For *C*_max_ and AUC_0–tlast_ of TU, DHTU, testosterone and DHT, parametric point estimates of the true ratio ‘test/reference’ of geometric least-squares means with their 90% confidence intervals (CIs) derived from the analysis of variance (anova) on log_e_-transformed values (multiplicative model) were calculated. The effects included in the anova model used were, respectively: sequence, subject within sequence, period, treatment and treatment × period (partial).

Effects were considered statistically significant when *P* ≤ 0·05 (two-tailed). The acceptance range was 0·80–1·20. A meal effect was considered absent if the 90% CI for *C*_max_ and AUC for testosterone and DHT were fully contained within the acceptance range of 0·80–1·20. Data pertaining to TU and DHTU were considered to be supportive.

For subjects where concentrations (of any analyte) throughout the sampling period were below the LLOQ, a value of one-half of the LLOQ was substituted for *C*_max_ and a value representing the AUC resulting from a concentration of one-half of the LLOQ during 1 h was substituted for AUC. For *t*_max_, classical hypothesis testing was performed. Point estimates of and nonparametric 95% CIs for median differences were calculated using the method of Walsh averages.^13^

## Results

### Subjects

All 24 randomized subjects completed the study. Mean age, weight, height and body mass index were 59 ± 4·2 years (range 50–65 years), 72·9 ± 9·9 kg (range 54·1–89·2 kg), 163·5 ± 5·9 cm (range 152–174 cm) and 27·2 ± 3·1 kg/m^2^ (range 20·1–32·1 kg/m^2^), respectively. All subjects were Caucasian women. Three subjects took concomitant medication (naproxen, acetyl salicylic acid, ibuprofen and articaine). Mean testosterone levels at inclusion were 1·8 ± 0·62 nmol/l(range 0·7–3·5 nmol/l).

During and after exposure to oral TU combined with meal A or meal D, 12 out of 24 women experienced adverse events (AEs), in five subjects these were classified as being drug related. After combination of oral TU application and meal B, nine of 24 women (38%) showed AEs and after treatment with oral TU and meal C, eight of 24 subjects (33%) reported an AE. No serious AEs or tolerability concerns were noted and all events reported were mild or moderate. AEs reported with a prevalence of > 10% were headache in 12 subjects (50%), diarrhoea in three subjects (13%) and fatigue in three subjects (13%). No findings of clinical relevance were indicated by electrocardiogram (ECG), physical examination, vital signs or laboratory investigations as assessed on the day before first dosing and 9 days after the last dose.

### Meals

Table 1 shows that the calculated amount of lipids does not differ substantially from the measured amount of lipid, except in meal D (50 g *vs.* 44 g). No remarkable differences between the calculated and actual total energetic values were seen, except for meal C (430 kcal *vs.* 474 kcal), but this difference was still less than 10%.

**Table 1:** Results of the food analysis compared with the calculated contents

Analysis	Meal A	Meal B	Meal C	Meal D
Total lipids (g)	0·61	5·43	18·76	44·09
Protein (g)	13·3	17·3	18·2	42·6
Total carbohydrates (g)	42·4	24·6	57·1	65·2
Total energetic value (kcal/kJ)	230/991	220/897	474/1992	837/3509
Calculated total lipids (g)	< 1	5	20	50
Calculated kcal	215	215	430	850

### Serum concentrations and pharmacokinetic parameters

**Fig. 1:**
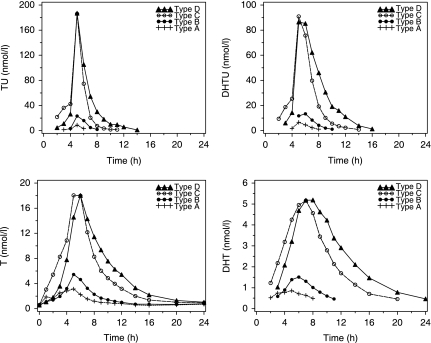
Geometric mean concentration *vs.* time curves for TU, DHTU, testosterone (T) and DHT after single oral administration of 80 mg TU. All curves based on *n* = 24 subjects.

The results of the serum concentrations of TU, DHTU, testosterone and DHT after the different type of meals are shown in Fig. 1. In general, meals A and B resulted in low exposure of all analytes. In many instances and on many time points, the serum concentrations were below or close to the LLOQ. Meals C and D resulted in much higher exposure for all analytes. Table 2 summarizes the calculated pharmacokinetic parameters and the results of the statistical analyses.

**Table 2:** Effect of food composition on the pharmacokinetics of TU, DHTU, testosterone and DHT after single oral administration of 80 mg TU

Parameter (units)	Meal A	Meal B	Meal C	Meal D
TU				
*C*_max_ (nmol/l)	20·2 (181)	47·8 (124)	372 (84·5)	382 (87·7)
*t*_max_ (h)	5·0 (2·0–11·0)	5·0 (2·0–7·0)	5·0 (2·0–7·0)	5·0 (2·0–12·0)
AUC_0–tlast_ (nmol h/l)	41·7 (176)	103 (149)	848 (53·3)	1050 (46·7)
DHTU				
*C*_max_ (nmol/l)	10·4 (160)	22·2 (91·0)	150 (53·7)	174 (56·2)
*t*_max_ (h)	5·0 (3·0–12·0)	5·0 (4·0–8·0)	5·0 (2·0–8·0)	6·0 (2·0–12·0)
AUC_0–tlast_ (nmol h/l)	27·1 (192)	66·8 (116)	479 (39·9)	677 (34·7)
Testosterone				
*C*_max_ (nmol/l)	4·65 (80·8)	7·10 (70·0)	27·3 (44·7)	27·0 (51·6)
*t*_max_ (h)	3·0 (1·0–11·0)	5·0 (1·0–8·0)	5·0 (2·0–7·0)	6·0 (2·0–12·0)
AUC_0–tlast_ (nmol h/l)	30·7 (59·9)	43·5 (48·2)	146 (30·6)	154 (32·2)
DHT				
*C*_max_ (nmol/l)	1·50 (65·8)	1·89 (53·7)	6·67 (45·7)	6·74 (49·3)
*t*_max_ (h)	3·0 (1·0–12·0)	6·0 (1·0–10·0)	7·0 (3·0–9·0)	7·0 (4·0–14·0)
AUC_0–tlast_ (nmol h/l)	7·62 (78·9)	11·0 (73·8)	49·5 (42·0)	57·9 (38·7)

Presented are geometric means (geometric CV%), except for *t*_max_: median (min − max).

*n* = 24 except for treatment type A, where *n* = 22 for TU and DHT and *n* = 23 for DHTU.

Values of *C*_max_ increased with increasing lipid content. However, increasing the lipid content beyond 19 g (meal D, 44 g) did not increase the *C*_max_ further for any of the analytes. For the AUC, increasing the lipid contents from < 1 to 5, 19 or 44 g resulted in an ongoing increase in AUC for TU and DHTU. However, for testosterone and DHT no further increase was seen when lipid content > 19 g was given. Lipid content did not have an effect on the *t*_max_ of TU and DHTU; however, for testosterone and DHT a low lipid content decreased *t*_max_ from 5 to 3 h, and increasing the lipid content tended to delay *t*_max_ from 3 to 6 h.

### Bioequivalence testing

Meals A and B were designed to differ only with regard to the total amount of lipids (< 1 g *vs.* 5 g) and not the amount of calories. There was a large difference in the total amount of lipids between these two meals, as well as a significant difference between the observed *C*_max_ and AUC for all analytes. Consequently, ingestion of similar amounts of calories with different lipid amounts led to a different extent of exposure.

As is already clear from the pharmacokinetic profiles, there were significant food effects observed for all analytes on comparing AUC and *C*_max_ of meal A *vs.* meal C and of meal B *vs.* meal C. Comparing meals C and D by testing for testosterone and DHT showed that these were bioequivalent for AUC of testosterone and *C*_max_ of DHT but indeterminant for *C*_max_ of testosterone and AUC of DHT (Table 3).

**Table 3:** Bioequivalence testing on the main analytes for the comparison of different meal compositions with meal C (reference)

Analyte	Parameter (units)	Point estimate	90% CI	Conclusion*
*Meal A (test) vs. meal C (reference)*				
Testosterone	*C*_max_ (nmol/l)	0·17	0·14–0·21	Not bioequivalent
	AUC_0–tlast_ (nmol h/l)	0·21	0·18–0·25	Not bioequivalent
DHT	*C*_max_ (nmol/l)	0·22	0·18–0·27	Not bioequivalent
	AUC_0–tlast_ (nmol h/l)	0·15	0·12–0·19	Not bioequivalent
*Meal B (test) vs. meal C (reference)*				
Testosterone	*C*_max_ (nmol/l)	0·26	0·21–0·33	Not bioequivalent
	AUC_0–tlast_ (nmol h/l)	0·30	0·25–0·35	Not bioequivalent
DHT	*C*_max_ (nmol/l)	0·29	0·24–0·36	Not bioequivalent
	AUC_0–tlast_ (nmol h/l)	0·23	0·18–0·29	Not bioequivalent
*Meal D (test) vs. meal C (reference)*				
Testosterone	*C*_max_ (nmol/l)	0·99	0·79–1·25	Indeterminant
	AUC_0–tlast_ (nmol h/l)	1·06	0·89–1·25	Bioequivalent
DHT	*C*_max_ (nmol/l)	0·99	0·80–1·22	Bioequivalent
	AUC_0–tlast_ (nmol h/l)	1·16	0·90–1·49	Indeterminant

Bioequivalent: the 90% confidence interval (CI) is inside the acceptance range of 0·80–1·25. Not bioequivalent: the 90% CI is outside the acceptance range and the effect is statistically significant (*P* = 0·05). Indeterminant: the 90% CI is outside the acceptance range and the effect is not statistically significant (*P* > 0·05).

## Discussion

In this food interaction study a single oral dose of 80 mg TU was administered to postmenopausal women after ingestion of various types of meals. It was demonstrated that the amount of lipids in a meal considerably influences the bioavailability of oral TU. A ‘normal’ meal (meal C) increased serum testosterone to a similar extent as a ‘fatty’ meal (meal D). A meal with a low calorie amount and low lipid content also led to a low exposure of testosterone and its precursor (TU) or metabolites (DHTU, DHT).

Postmenopausal women were selected for this study because they already have low endogenous testosterone levels and do not need a long-term washout period from previous androgen therapy, as would be the case in hypogonadal males, or downregulation with a GnRH agonist, as would be the case in healthy male volunteers (either of which would have ethical and pathophysiological implications). The most common daily dose of oral TU prescribed in hypogonadal men is 80 mg in the morning and 80 mg in the evening. Therefore, the regimen chosen for this study was 80 mg TU as an oral single dose, given with four different kinds of food.

In the vast majority of cases testosterone supplementation is prescribed in men rather than women. Although it is known that there are sex differences in metabolic clearance rates and distribution volumes of testosterone,^14^ this is not considered relevant for an investigation of the effects of food composition on testosterone absorption in a crossover study, where each subject acted as her own control. Moreover, food uptake and lymphatic absorption of lipophilic food ingredients are both not known to be different between men and women. It should be realized, however, that because of the sex differences in testosterone metabolism, the results obtained in this study in postmenopausal women on absolute testosterone serum levels cannot automatically be applied to hypogonadal men.

By comparing a meal with similar amounts of calories (meal A/B) but different amount of lipids (< 1 g *vs.* 5 g) it was shown that bioavailability increases with increasing amounts of lipids. For example, the AUC for TU was 41·7 nmol h/l(meal A) *vs.* 103 nmol h/l(meal B) and for testosterone the corresponding figures were 30·7 nmol h/l*vs.* 43·5 nmol h/l, respectively. It is clear that not all analytes were similarly affected by the amount of lipids in the meal. However, from these data it is also clear that there seems to be a maximum to the amount of lipids needed to get efficient absorption of TU after oral administration. There were no statistical differences seen in exposure after a meal with 19 g or 44 g of lipids, indicating that a lipid amount of 19 g is already sufficient for efficient uptake and that increasing the amount of lipid over 19 g does not lead to a higher exposure. This is an important finding because testosterone levels need to be restored to physiological levels, and during oral administration of TU they do not increase indefinitely with increasing amounts of fat.

Meals A/B and C/D not only differed in amounts of calories and lipids but also in consistency because meals A and B were liquid and meals C and D solid. To what extent this difference contributed to the difference in exposure needs to be further investigated, but it is clear that a liquid meal consisting only of yoghurt and milk, with at most 5 g of lipid, is not sufficient for adequate absorption of oral TU.

The effects of different vehicles on absorption of oral TU have been tested in the rat. It was proven that oral TU dissolved in long-chain fatty acid esters was better absorbed by the lymphatic system than oral TU dissolved in medium- or short-chain fatty acids. However, the solubility of oral TU in some of these oils was limited.^15^ During the initial development in the 1970s, oleic acid was chosen as the best compound, combining solubilization and absorption. Recently, the solvent was changed to castor oil, enabling better storage conditions.^4^ When the testosterone ester is orally administered together with a lipid, part of the compound is incorporated in chylomicrons formed during lipid digestion in the intestine and coabsorbed with the lipid into the lymphatic system. During absorption, TU is partly reduced to DHTU, which is also absorbed by the lymphatic system. From the lymphatic system, TU and DHTU are then released into the systemic circulation by the thoracic duct. Subsequent hydrolysis of the ester liberates testosterone and DHT, which then follow exactly the same pathway in the body as the endogenous hormones. In a validated dog model, it has been demonstrated that lymphatic absorption is responsible for most of the testosterone entering the systemic circulation after administration of oral TU.^16^

In general, lymphatic absorption is known to be enhanced by food, specifically by lipids. This has been demonstrated in a validated dog model with halofantrine,^17^ as well as in humans with experimental^10^ and registered formulations of oral TU.^11^ In the last study, two capsules of oral TU (80 mg) were administered in postmenopausal women as a single dose with food (460 kcal and 23 g lipids) *vs.* administration of the same without food. Administration of oral TU with food resulted in a *C*_max_ of testosterone of 37 nmol/l as compared to 2·4 nmol/l in the fasted group (16-fold difference), whereas the corresponding values for AUC were 195 *vs.* 19 nmol h/l (10-fold difference), respectively. In the current study it was found that a ‘normal meal’ (with a lipid amount of 19 g) resulted in a marked increase in testosterone levels and that a ‘fatty meal’ (with a lipid amount of 44 g) did not result in a further increase in testosterone absorption. These findings suggest that, with the ‘normal’ meal, the lymphatic absorption capacity for testosterone undecanoate has already reached its maximum and that a further increase in the dietary fat does not further enhance lymphatic absorption. The findings from the current study, however, also suggest that a low-calorie breakfast, for example a breakfast consisting of fruit and juice only, is probably not enough for adequate lymphatic absorption of oral TU.

The American Heart Association (AHA) recommends that up to 30% of the total caloric intake should come from fat.^18^ In addition to being a major energy source, fat is also essential for isolation, vitamin production and many immunological and metabolic processes. For an average 2500 kcal diet this means that 750 kcal should come from fat, which corresponds to a daily total of 83 g of fat (the caloric value of fat is 9 kcal/g). This AHA recommendation of 83 g is more than double the 38 g (2 × 19 g) of fat that is needed for adequate absorption of oral TU per day when taken in a regimen of 2 × 2 capsules per day. From this calculation it also becomes clear that a ‘normal’ meal contains enough fat for adequate absorption of oral TU.

The current study also revealed that with oral TU there is a considerable interindividual variability in exposure to testosterone: 50% for *C*_max_ and 40% for AUC. These interindividual differences may, in individual cases, result in subnormal serum testosterone levels. This may be related to the variable absorption of TU due to the oral administration route. Therefore, subjects in whom testosterone is not adequately supplemented with a standard dose of oral TU are advised either to increase their daily dosage or to switch to an alternative testosterone formulation. However, for meals C and D the bioavailability per individual in relation to the other individuals appears fairly consistent. For example, 20 of the 24 women had a serum testosterone AUC below or above the median AUC for meal C as well as meal D. Thus, the lower or higher bioavailability of testosterone of an individual after oral administration of TU seems to be fairly reproducible when administered after meals C and D.

From placebo-controlled clinical trials with objective clinical end-points, it can be concluded that, overall, the total testosterone exposure in subjects treated with oral TU is probably sufficient because the magnitude of effects is not different from other testosterone preparations. For example, in a recent study with oral TU 160 mg/day,^19^ the increase in muscle mass and reduction in fat mass were similar to those previously reported for testosterone injection,^20^ patch^21^,^22^ or gel^23^,^24^ preparations at standard doses. The clinical equivalence of oral TU with gel or patch formulations was recently confirmed by a consensus paper that has been adopted by several scientific bodies including the International Society of Andrology.^25^ The pharmacokinetic behaviour as observed with oral TU can sometimes make it difficult to monitor treatment adequacy in an individual by checking serum testosterone levels. In such cases it is recommended to monitor the adequacy of the dose by assessment of the clinical response to treatment, preferably approximately 3 months after start of therapy, when the patient is usually seen back by the physician.

From this well-controlled pharmacokinetic study it can be concluded that lipid content in food influences absorption of oral TU. A liquid meal with, at most, 5 g of lipid is not sufficient for adequate absorption. It is, however, also not necessary to administer oral TU with a ‘fatty’ meal; a ‘normal’ meal containing approximately 19 g of lipid efficiently increases serum testosterone levels after oral administration of TU capsules.
